# The Emerging Role of Postbiotics Against Microplastics-Induced Inflammatory Bowel Disease

**DOI:** 10.3390/foods15213899

**Published:** 2026-10-30

**Authors:** Nader Khani, Shadi Rajabi, Mohammadreza Ziavand, Roya Abedi-Soleimani, Seyed Mohamad Javad Shokouhian, Samira Saedi, Aziz Homayouni-Rad

**Affiliations:** 1 Student Research Committee, Tabriz University of Medical Sciences, Tabriz 5166614766, Iran; naderxani1996m@gmail.com (N.K.);; 2 Department of Food Science and Technology, Faculty of Nutrition & Food Sciences, Tabriz University of Medical Sciences, Tabriz 5166614766, Iran; 3 Department of Food Hygiene and Quality Control, Faculty of Veterinary Medicine, Urmia University, Urmia 5756151818, Iran; 4 Faculty of Veterinary Medicine, Karaj Branch, Islamic Azad University, Karaj 3149968111, Iran

**Keywords:** probiotics, microbial dysbiosis, inflammation, gut barrier function, immunomodulation, microplastics

## Abstract

Microplastics (MPs), particularly those composed of polystyrene (PS), pose significant toxicological risks to humans, especially through oral exposure. Increasing evidence suggests that MPs contribute to the development of inflammatory bowel disease (IBD). Postbiotics have recently gained attention for their protective effects against various chemical pollutants, including MPs. They can alleviate MP-induced IBD through several mechanisms, such as anti-inflammatory activity, immunomodulatory effects, inhibition of pathogenic bacterial growth, and maintenance of intestinal barrier integrity. This review highlights the definitions of postbiotics, as well as their biological roles in the prevention and treatment of microplastics-induced inflammatory bowel disease.

## 1. Introduction

MPs are plastic particles smaller than 5 mm in diameter [1]. Their extensive distribution in terrestrial and aquatic ecosystems, coupled with their high bioavailability, has resulted in their detection in a wide range of food products. MPs can enter the human food chain through several pathways, including the consumption of contaminated animal-derived foods and exposure during various stages of food processing, particularly through plastic packaging and wrapping materials [2,3,4]. The most common MPs encountered by humans include polystyrene (PS), polyethylene (PE), polypropylene (PP), polyurethane (PU), polyethylene terephthalate (PET), polyvinyl chloride (PVC), and polyamide (PA). Multiple studies have reported MP contamination in diverse food items such as honey, seafood, and table salt and beverages such as bottled water and beer [5,6,7]. Once ingested, MPs pass through the oral cavity, esophagus, stomach, and intestines, making the gastrointestinal (GI) tract the first site where toxicological effects are typically observed.

A growing body of evidence links MPs to IBD, including Crohn’s disease and ulcerative colitis [8]. MPs can disrupt the gut microbiota by reducing beneficial microorganisms such as *Lactobacillus* spp. and *Bifidobacterium* spp., while promoting the proliferation of pro-inflammatory bacteria, ultimately leading to gut dysbiosis a well-established trigger of IBD [9]. Their small size, irregular surfaces, and abrasive nature allow MPs to irritate the intestinal lining and increase intestinal permeability, facilitating the translocation of bacteria and toxins into the bloodstream. This breach activates immune cells within the gut-associated lymphoid tissue (GALT), resulting in persistent immune stimulation and chronic inflammation, a hallmark of IBD [10]. IBD itself is characterized by long-term inflammation of the digestive tract, presenting primarily as Crohn’s disease which may affect any part of the GI tract or ulcerative colitis, which is restricted to the colon and rectum [11]. Clinical manifestations typically include abdominal pain, diarrhea, rectal bleeding, weight loss, and fatigue, occurring in cycles of flare-ups and remission [12].

In recent years, the application of postbiotics has gained substantial interest due to their potential to enhance animal welfare, improve human health, and contribute to environmental sustainability. Postbiotic consumption provides several benefits, particularly through supporting digestive function and modulating immune responses. Numerous components of postbiotics have shown effectiveness in mitigating colitis in murine models by modulating the gut microbiota, suppressing excessive immune activation, and strengthening intestinal barrier integrity. These compounds promote the growth of beneficial microorganisms while reducing populations of pathogenic species [13]. Postbiotics are considered safe and advantageous across all life stages, including childhood, elderly populations, pregnancy, and lactation. Understanding the role of MPs in the development of IBD is essential for exploring postbiotic-based therapeutic strategies aimed at restoring gut homeostasis. Therefore, this review examines the critical role of gut dysbiosis in IBD and outlines how postbiotic compounds can effectively modulate the altered microbial ecosystem [14]. Ultimately, the review highlights the definitions of postbiotics and emphasizes their biological roles in the prevention and treatment of microplastics-induced inflammatory bowel disease.

## 2. Health Implications and Pathways of Microplastics Entering the Human Body

Microplastics (MPs) are distributed pervasively across ecological systems, and several empirical studies have confirmed their presence in human feces, indicating that MPs can enter the human body primarily through alimentary and waterborne routes [15,16]. Previous research has identified three principal pathways through which MPs infiltrate the human organism: ingestion, inhalation, and dermal exposure (Figure 1) [17].

Ingestion is considered the primary route of exposure, occurring through the consumption of contaminated food and water sources such as seafood, bottled water, table salt, fruits, and vegetables that have accumulated or absorbed plastic particles. Inhalation of airborne MPs from dust, textile fibers, household environments, or industrial emissions also contributes to exposure. Inhaled particles can reach the respiratory tract and may be subsequently swallowed, allowing them to enter the GI system [18]. Dermal exposure through contact with personal care products or contaminated surfaces is also possible; however, this pathway is considered minor compared with ingestion and inhalation. Once ingested, MPs can accumulate in the GI tract, alter the gut microbiota, impair intestinal barrier integrity, and trigger local or systemic inflammatory responses. Hence, ingestion remains the dominant and most biologically significant pathway for intestinal exposure to MPs [19]. In experimental research, intestinal exposure to MPs is typically modeled via oral routes, including gavage, drinking-water supplementation, or incorporation into animal feed, to closely simulate human ingestion patterns [20]. In vivo studies generally administer MPs at doses ranging from 1 to 100 mg/kg body weight per day, whereas drinking-water exposure models use concentrations between 0.0025 and 0.125 mg/mL. In vitro studies employing intestinal cell lines commonly apply MP concentrations ranging from 0.01 to 1000 µg/mL to assess cytotoxicity, oxidative stress, inflammatory signaling, and barrier dysfunction [21].

Microplastics exert widespread toxic effects on multiple body systems, including the hepatic, renal, nervous, and pulmonary systems [22]. In the liver, absorbed MPs or their associated chemical additives can accumulate within hepatic tissue, where they induce hepatocellular damage, disrupt lipid metabolism, and trigger inflammatory responses through mechanisms involving oxidative stress and mitochondrial dysfunction [23]. Within the renal system, MPs that enter systemic circulation may reach the kidneys, impair glomerular filtration, promote tubular injury, and elevate oxidative and inflammatory biomarkers, ultimately contributing to renal dysfunction [24]. In the nervous system, MPs are capable of crossing the blood–brain barrier or influencing brain function indirectly via gut–brain axis dysregulation. These effects can result in neuroinflammation, alterations in neurotransmitter levels, and measurable behavioral impairments [25]. In the pulmonary system, inhaled MPs can deposit along the respiratory tract, activating immune responses, promoting oxidative stress, and inducing fibrosis-like pathological changes in lung tissue. Collectively, MPs function as persistent environmental pollutants capable of eliciting systemic toxicity through inflammation, oxidative damage, and immune dysregulation across multiple organ systems [26].

## 3. MPs-Induced IBD

MPs can induce marked dysbiosis within the intestinal microbiota. Under physiological conditions, the balance between proliferation and differentiation of intestinal stem cells is tightly regulated to ensure proper tissue homeostasis and prevent uncontrolled cell expansion. Excessive production or depletion of specialized epithelial cells such as Paneth and goblet cells may contribute to inflammatory disorders including ulcerative colitis and Crohn’s disease, and may even predispose to malignancies. Numerous investigations have shown that exposure to PE MPs results in a significant reduction in goblet cell numbers, along with an increase in both the quantity and depth of colonic epithelial crypts. Genes associated with intestinal stem cell markers are markedly upregulated, and key regulators of proliferation such as c-myc and proliferating cell nuclear antigen (PCNA) show substantial elevation within the colonic crypts [27]. The Notch signaling pathway, a major determinant of intestinal stem cell fate and goblet-cell differentiation, has been implicated as a critical mediator of these effects. Hyperactivation of Notch signaling in intestinal organoids exposed to PE MPs disrupts normal crypt proliferation and differentiation, indicating that the colonic epithelium enters a state of heightened proliferative activity following MP exposure. These findings highlight a disruption in the balance between self-renewal and differentiation of intestinal stem cells, ultimately disturbing intestinal homeostasis a disturbance strongly associated with increased susceptibility to intestinal diseases. IBD is recognized as a chronic inflammatory condition triggered by environmental factors, and its onset has been closely linked to such disruptions in epithelial homeostasis [28]. Clinical findings further reveal that individuals with IBD exhibit significantly higher concentrations of PE MPs in their feces compared with healthy populations. Experimental studies show that exposure to PE MPs accelerates colitis onset, causing rapid weight loss, diarrhea, hematochezia, macroscopic tissue injury, histopathological alterations, and intensified inflammatory responses. In murine colitis models, MPs also aggravate hepatic inflammation and pathological injury. Together, these findings support the notion that the delicate balance of self-renewal and differentiation in colonic epithelial cells is vulnerable to disruption by MPs, potentially increasing susceptibility to environmentally induced intestinal disorders [29].

Microbiome-related alterations also play a key role. Overgrowth of *Staphylococcus* species has been associated with IBD due to superantigen-driven inflammatory responses. Patients with ulcerative colitis exhibit significantly reduced *Parabacteroides* levels compared with healthy individuals [30]. Reduced *Akkermansia* abundance correlates with impaired intestinal barrier integrity, elevated inflammation, and an increased risk of weight gain. PE MP exposure elevates levels of multiple cytokines including IL-1α, G-CSF, IL-2, IL-5, IL-6, IL-9, and IP-10 increases the proportions of Th1 and Th17 cells. IL-6, a central regulator in both sporadic and IBD-related inflammation, is particularly responsive to MP exposure. PE MPs may also expand *Staphylococcus* populations, contributing to increased IL-1 levels. G-CSF promotes chronic inflammation by driving neutrophil recruitment. Beneficial species such as *Bacteroides fragilis* promote regulatory T cell (Treg) differentiation and suppress Th17 expansion, while *Clostridium*-mediated induction of Treg cells is essential for maintaining gut immune homeostasis. Thus, PE MPs may disturb the Th17/Treg balance by altering gut microbiota diversity [31]. The gut microbiota also activates protective mechanisms that modulate mucosal immune responses, including Toll-like receptor (TLR) signaling pathways. TLRs, particularly TLR4, are critical regulators of mucosal inflammation. Elevated TLR4 expression has been observed in the colonic mucosa of pediatric IBD patients. Pro-inflammatory transcription factors AP-1 and IRF5, which operate downstream of TLR4, are significantly upregulated in experimental models exposed to MPs. Activation of TLR4/AP-1 and TLR4/IRF5 pathways has been shown to play a major role in intestinal inflammation, with markedly increased TLR4, AP-1, and IRF5 expression in MP-exposed groups compared with controls [32]. Collectively, these findings suggest that PE MPs disrupt gut microbiota composition, alter microbial abundance and diversity, and consequently modulate cytokine secretion and the proportions of Th1, Th17, and Treg cells. These immune alterations are hypothesized to trigger intestinal inflammation through activation of TLR4-mediated signaling pathways (Figure 2).

Recent research has examined the ability of gut microbiota to activate the aryl hydrocarbon receptor (AhR) following exposure to MPs. Many environmental contaminants are known to trigger pro-inflammatory responses across different cell types through AhR-mediated pathways [33]. The gut microbiota produces several tryptophan-derived AhR ligands—including indole-3-acrylic acid, indole-3-acetic acid, tryptamine, indole-3-aldehyde, and indole-3-acetaldehyde—that play essential roles in maintaining intestinal epithelial integrity and regulating the development, function, and homeostasis of key mucosal immune cells and mediators [34]. Notably, individuals with IBD display significantly reduced production of these microbial AhR ligands. Following PE MP exposure, AhR activity in the gut is generally diminished compared with controls. In C57BL/6 mice, PE MPs induced expansion of mucosal and mucin-secreting regions and increased transcript levels of Muc2, Vil1, and Chga in the colon, indicating disrupted epithelial differentiation [35]. Upregulation of Ocln and F11r further suggests alterations in epithelial junctional integrity. PE microplastic exposure also elevated expression of Ifng and Il6, promoting a pro-inflammatory environment within the colon. Significant shifts in intestinal immune profiles were observed, including altered frequencies of CD4^+^ and CD8^+^ T lymphocytes, dendritic cells, and inflammatory monocytes in the proximal small intestine; reduced NK cell numbers in the distal small intestine; and changes in anti-inflammatory macrophage populations in the colon [36]. Moreover, exposure to polyethylene beads decreased the abundance of beneficial *Lactobacillus* spp., while increasing the frequency of *Rhodospirillales*, indicating substantial dysbiosis.

Collectively, these findings demonstrate that polyethylene microplastics disrupt gut microbial composition, suppress AhR-mediated protective signaling, alter immune cell distribution, and impair epithelial barrier homeostasis all of which contribute to the development and progression of IBD [10,37].

The introduction of MPs into the human GI system has been shown to significantly alter the composition of microbial volatile metabolites, which can be distinguished by five major discriminant volatile organic compounds. Following PE MP exposure, a notable increase in the concentration of skatole (3-methylindole) was observed, while the levels of three hydrocarbons and one alcohol markedly decreased [38]. Skatole is a microbial metabolite derived from tryptophan and absorbed by intestinal epithelial cells. Its formation involves the decarboxylation of indole-3-acetic acid, a process mediated by gut microbiota residing in both the small intestine and colon [39]. Skatole is a microbial metabolite derived from tryptophan and absorbed by intestinal epithelial cells. Its formation involves the decarboxylation of indole-3-acetic acid, a process mediated by gut microbiota residing in both the small intestine and colon [40]. Our analysis revealed an increased abundance of *Desulfovibrio*, which may enhance skatole production and partially explain the elevated levels observed following PE MP exposure. Notably, skatole is typically undetectable in the large intestine during healthy fermentation; however, significant concentrations have been reported in the GI tract of individuals with IBD. The substantial rise in 3-methylindole after PE MP exposure suggests that GI dysregulation may be driven, in part, by alterations in microbial composition. Further investigation into this mechanism is warranted [41,42]. A wide range of therapeutic approaches is available for IBD management, including conventional pharmacological treatments, dietary interventions, and advanced biologic therapies. In recent years, postbiotics bioactive compounds produced by beneficial microorganisms have emerged as a promising complementary strategy. Postbiotics can modulate immune responses, enhance intestinal barrier integrity, and promote overall gut health, offering a supportive therapeutic avenue alongside established IBD treatments [43,44].

## 4. Concept of Postbiotics

According to the International Scientific Association for Probiotics and Prebiotics (ISAPP), a “postbiotic” is defined as a preparation of non-living microorganisms and/or their components that confers health benefits to the host [45,46,47,48]. Postbiotics occur naturally in a variety of fermented foods including kefir, kimchi, sauerkraut, tempeh, yogurt, and certain pickled products as well as within the human GI tract. The major classes of postbiotics include organic acids, short-chain fatty acids (SCFAs), tryptophan (Trp) metabolites, exopolysaccharides (EPS), and bacteriocins [49,50,51]. Their beneficial effects may be direct through interactions with host cells or indirect, by promoting the growth of beneficial microorganisms and suppressing harmful species. As with prebiotics, the specific biological effects depend heavily on the microbial strain and the metabolic products it generates. Among these compounds, SCFAs exhibit particularly strong anti-inflammatory and antioxidant activities [52,53,54,55,56,57,58,59,60].

Postbiotics display a high degree of biological specificity, meaning that their functions depend on the producing microorganism, the metabolic environment, and the physiological state of the host. Each bacterial strain produces a distinct profile of metabolites such as SCFAs, peptides, or EPS that interact with specific host receptors or intracellular pathways [61]. For example, butyrate produced by *Faecalibacterium prausnitzii* primarily targets colonocytes and regulates anti-inflammatory responses via GPR41 and GPR43 receptors, whereas peptides derived from *Lactobacillus* species can modulate immune cell differentiation and cytokine expression. This strain-specific activity highlights that not all postbiotics exert identical effects, even if they belong to similar chemical categories [62]. Additionally, the host’s microbiota composition, dietary patterns, and overall health status can influence the specificity and efficacy of postbiotics, supporting the need for personalized or targeted therapeutic applications [63]. The biological activity of postbiotics is also strongly influenced by the method used to inactivate the parental microorganisms. Different inactivation approaches including heat treatment, high-pressure processing, ultraviolet or gamma irradiation, chemical agents, and sonication—modify microbial structures and biochemical components in distinct ways [64]. The biological activity of postbiotics is also strongly influenced by the method used to inactivate the parental microorganisms. Different inactivation approaches including heat treatment, high-pressure processing, ultraviolet or gamma irradiation, chemical agents, and sonication modify microbial structures and biochemical components in distinct ways [65]. Thus, the functional properties of postbiotics including stability, safety, and immune-modulating potential are closely dependent on the chosen inactivation method [66].

Postbiotics offer several safety advantages over probiotics because they consist solely of inanimate microbial cells or their metabolites, thereby eliminating risks of systemic infection, translocation, or uncontrolled replication in vulnerable individuals [67]. Unlike live probiotics, postbiotics cannot proliferate, acquire mutations, or transfer antibiotic resistance genes, making them safer for infants, elderly individuals, and immunocompromised patients. In addition, their chemical stability and predictable composition provide superior quality control, storage stability, and dosage standardization. These characteristics position postbiotics as a highly safe and reliable alternative to probiotics in both food products and clinical applications [68].

## 5. Postbiotics and Their Protective Effects Against MPs-Induced IBD

MPs induce IBD through several interconnected molecular pathways. After ingestion, MPs accumulate within the intestinal epithelium, compromise epithelial tight junctions, and increase intestinal permeability. This barrier disruption facilitates the translocation of bacterial components such as lipopolysaccharides (LPS), which activate TLRs particularly TLR4. Activation of TLR4 initiates downstream signaling through the MyD88 adaptor, promoting NF-κB and MAPK pathway activation and stimulating the production of pro-inflammatory cytokines including TNF-α, IL-6, and IL-1β [69]. These pathways promote the production of pro-inflammatory cytokines such as TNF-α, IL-6, and IL-1β, which further amplify intestinal inflammation. Additionally, microplastics can generate reactive oxygen species (ROS), causing oxidative stress and mitochondrial dysfunction, which exacerbate epithelial injury and immune dysregulation. Collectively, these molecular events contribute to chronic intestinal inflammation characteristic of IBD [70].

Postbiotics counteract MPs-induced IBD through multiple protective mechanisms that restore intestinal homeostasis and inhibit inflammation. One of their primary actions is reinforcement of the intestinal barrier. SCFAs, especially butyrate, upregulate tight junction proteins such as occludin, claudins, and ZO-1, reducing the permeability induced by MPs. Strengthening the epithelial barrier prevents LPS translocation and subsequent inflammatory activation [71]. Postbiotics also modulate gut microbiota composition by inhibiting pathogenic bacteria and promoting beneficial commensals, thereby preventing MP-induced dysbiosis. At the signaling level, postbiotics inhibit NF-κB and MAPK pathways, reducing the expression of pro-inflammatory cytokines including TNF-α, IL-6, and IL-1β. They also enhance IL-10 production and activate PPAR-γ, which downregulates inflammatory gene transcription. Additionally, postbiotics possess antioxidant properties that decrease ROS formation and improve mitochondrial function, collectively protecting intestinal epithelial cells from MP-induced oxidative damage [72,73]. Postbiotics also strengthen the intestinal barrier through mucus enhancement and immune support. SCFAs such as butyrate, acetate, and propionate increase the expression of tight junction proteins and stimulate mucus secretion by goblet cells, forming a protective layer that limits pathogen and particle penetration. They further enhance production of antimicrobial peptides and secretory IgA, reinforcing mucosal immunity. Through these actions, postbiotics prevent MP-induced epithelial disruption and help maintain gut homeostasis, reducing chronic intestinal inflammation linked to IBD [74,75].

Immunomodulation is another key protective function of postbiotics. MPs activate innate immunity via TLRs and elevate pro-inflammatory cytokines such as TNF-α, IL-6, and IL-1β. Postbiotics including SCFAs and microbial polysaccharides suppress NF-κB and MAPK activation, thereby reducing cytokine overproduction [71]. They promote Treg differentiation and enhance anti-inflammatory cytokine secretion, including IL-10 and

TGF-β. Postbiotics also reduce macrophage and neutrophil activation, diminishing oxidative stress and tissue injury in the intestinal mucosa. These immunomodulatory mechanisms restore immune balance, limit chronic inflammation, and protect intestinal tissues from MP-induced damage [76]. Finally, postbiotics play a crucial role in restoring gut microbiota composition disrupted by MP exposure. MPs reduce beneficial populations such as *Lactobacillus* spp. and *Bifidobacterium* spp. while supporting pathogenic bacteria that aggravate inflammation [44]. Postbiotics including SCFAs, exopolysaccharides, and bacteriocins reestablish microbial homeostasis by promoting beneficial bacteria and suppressing pathogens. SCFAs, particularly butyrate, nourish colonocytes and lower intestinal pH, thereby inhibiting pathogenic growth [77]. Postbiotics also enhance microbial diversity and facilitate production of anti-inflammatory metabolites that stabilize gut physiology. Through these restorative actions, postbiotics counteract MP-induced dysbiosis, reduce inflammation, and support intestinal health in IBD conditions [78].

## 6. The Potential of Individual Postbiotics in Improving IBD

Individual postbiotics have attracted increasing attention for their potential role in supporting the treatment of IBD. These bioactive metabolites can exert targeted effects on gut immunity, inflammatory pathways, and mucosal integrity. By acting directly on cellular signaling mechanisms and strengthening epithelial barrier function, individual postbiotic compounds offer a more stable, controlled, and mechanistically precise alternative to traditional probiotics (Figure 3) [79]. Numerous studies have investigated these therapeutic effects (Table 1).

### 6.1. SCFA

SCFAs represent the most extensively studied category of postbiotics. They are synthesized by the gut microbiota through the fermentation of plant-derived polysaccharides such as oligofructose and inulin resulting in the production of acetate, propionate, and butyrate [92]. SCFAs are essential metabolites that help maintain intestinal homeostasis. One of their primary functions is lowering luminal pH, which inhibits the growth of pathogenic bacteria. Owing to their distinct chemical structures, each SCFA exerts unique physiological effects on human health. Butyrate is the most thoroughly investigated SCFA. It serves as the principal energy source for intestinal epithelial cells and plays a critical role in epithelial regeneration [93]. Butyrate is mainly produced by gut bacteria, particularly *Enterococcus faecalis* and *Enterococcus rectus*, via CoA-transferase or butyrate kinase pathways. Numerous studies have demonstrated a significant reduction in butyrate-producing bacteria in individuals with IBD compared with healthy controls [93]. Beyond its epithelial effects, butyrate possesses notable anti-tumor properties by downregulating the canonical Wnt signaling pathway and inhibiting proliferation of colon cancer cells through histone deacetylase inhibition, ultimately promoting apoptosis in neoplastic cells [94]. Beyond its epithelial effects, butyrate possesses notable anti-tumor properties by downregulating the canonical Wnt signaling pathway and inhibiting proliferation of colon cancer cells through histone deacetylase inhibition, ultimately promoting apoptosis in neoplastic cells [95]. Butyrate also exhibits strong immunoregulatory functions: it enhances the production of immunosuppressive cytokines, suppresses pro-inflammatory cytokines and their receptors, and reduces mucosal inflammation by inhibiting neutrophil-mediated immune responses and the formation of neutrophil extracellular traps. Propionate is another major SCFA that serves as a key energy substrate for intestinal mucosal cells [96]. It is generated through several bacterial fermentation pathways, including the succinate, acrylate, and propylene glycol pathways. Numerous studies have linked propionate to a variety of metabolic processes. For example, propionate can inhibit adipogenesis and reduce serum cholesterol levels, leading to decreased weight gain, improved insulin sensitivity, reduced appetite, and altered visceral fat distribution in overweight individuals. SCFAs can be administered as individual postbiotic compounds without the presence of live bacteria or prebiotics. However, purified SCFAs typically show reduced efficacy compared with postbiotic mixtures, largely due to the synergistic effects produced by multiple bioactive components within complex postbiotic preparations [97].

### 6.2. Bacteriocins

Bacteriocins are ribosomally synthesized antimicrobial peptides produced by many gut commensal bacteria particularly lactic acid bacteria that selectively inhibit or kill pathogenic and opportunistic species. In addition to their direct antimicrobial activity, bacteriocins and bacteriocin-producing strains can reshape microbial community structure, suppress pathogen overgrowth, and help restore the balanced microbiota that is frequently disrupted by microplastic exposure [98]. This community-level rebalancing is crucial, as microplastics have been shown in both animal and human studies to disturb microbiome composition, exacerbate colitis-like inflammation, and create ecological niches that support the expansion of proinflammatory or opportunistic species. Bacteriocins may protect against microplastic-induced IBD through several complementary pathways: (1) directly suppressing pathogens that flourish after microplastic injury and limiting pathogen-induced inflammation; (2) promoting beneficial symbionts either by eliminating competitors or through signaling that helps improve SCFA producers, which themselves strengthen the epithelial barrier and reduce inflammation; (3) modulating host immune responses—some bacteriocins or bacteriocin-producing strains reduce proinflammatory cytokine signaling and NF-κB activation in epithelial and immune cells; and (4) indirectly enhancing barrier integrity through increased mucin production and microbiota-mediated tight junction protein expression, which counteracts the barrier disruption caused by microplastics. Despite these promising mechanisms, research specifically evaluating purified bacteriocins rather than bacteriocin-producing probiotic strains in microplastic exposure models remains limited [74].

### 6.3. Trp

Numerous metabolites produced through microbial degradation of tryptophan serve as ligands for the AhR. AhR is a key transcription factor that regulates the expression of genes involved in xenobiotic metabolism, including dioxins and pharmaceutical compounds broken down by the cytochrome P450 enzyme system [99,100]. Microbial tryptophan-derived metabolites that act as AhR ligands are essential for protecting the intestinal mucosa from inflammatory damage. AhR activation directly enhances the transcription of several target genes, including IL-6, IL-22, and CYP1A1, the latter serving as a key feedback regulator of AhR signaling. AhR-mediated pathways form a central component of the immune response at mucosal barrier surfaces. They help maintain intestinal homeostasis by promoting epithelial regeneration, preserving barrier integrity, and regulating a diverse range of immune cell populations, including intraepithelial lymphocytes, Th17 cells, innate lymphoid cells, macrophages, dendritic cells, and neutrophils. Microbial metabolism of tryptophan produces multiple postbiotic metabolites including indole-3-acetate and indole-3-propionate which exhibit well-documented anti-inflammatory properties [101]. Consequently, reduced microbial tryptophan metabolism may negatively influence IBD pathophysiology [102]. Animal studies have shown that levels of indole-3-acetate and indole-3-propionate decline during IBD progression. Host-derived enzymes involved in tryptophan metabolism also play a role in modulating IBD severity. Indoleamine 2,3-dioxygenase 1 (IDO1), which catalyzes the conversion of tryptophan to kynurenine, is particularly important in regulating acquired immunity. IDO1 is markedly overexpressed both in the GI tract and systemically in individuals with active IBD, and its activity correlates negatively with circulating tryptophan levels and positively with C-reactive protein concentrations [103]. IDO1 is expressed by intestinal mononuclear cells, including T lymphocytes found within inflammatory infiltrates of the intestinal wall [104]. The immunosuppressive functions of IDO1 are multifaceted and include the suppression of CD8^+^ effector T cells and NK cells, alongside the enhancement of CD4^+^ Treg activity [105]. Patients with IBD typically exhibit elevated levels of tryptophan metabolites such as kynurenine and kynurenic acid, while showing reduced plasma tryptophan levels. Similar metabolic patterns are observed within the GI tract. Pro-inflammatory cytokines including IL-2, IL-6, and TNF-α promote the conversion of tryptophan into downstream metabolites by increasing IDO1 expression. Elevated IDO1 expression has been confirmed in both peripheral blood and colonic lymphocytes of IBD patients [106]. associated with intestinal inflammation. Approximately 1–2% of dietary tryptophan is converted to serotonin, with ~90% of total serotonin produced in enterochromaffin cells. Serotonin (5-HT) plays a central role in GI physiology, influencing enteric neuronal signaling, gut motility, vasodilation, secretion, and nutrient absorption [107,108]. The alterations observed in tryptophan metabolism and its microbial derivatives suggest that these pathways represent promising therapeutic targets. For example, administration of *Lactobacillus* strains capable of producing AhR agonists has been shown to reduce colonic inflammation in mouse models with genetically driven dysbiosis [109]. *Lactobacillus reuteri*, which produces the AhR agonist indole-3-lactic acid, can reprogram intraepithelial CD4^+^ T cells into CD4^+^CD8αα^+^ immunoregulatory cells [110]. In conditions characterized by excessive IDO1 activation as commonly observed in IBD microbial synthesis of AhR agonists may be reduced due to tryptophan depletion. In such cases, dietary tryptophan supplementation has been shown to ameliorate colitis symptoms in both murine and porcine inflammation models [111].

### 6.4. CFS

Cell-free supernatant (CFS), defined as the liquid fraction obtained from bacterial cultures after the removal of microbial cells, has emerged as a promising postbiotic candidate for alleviating microplastic-induced IBD. In the context of MPs-associated intestinal injury, CFS can counteract multiple pathological mechanisms.

First, CFS strengthens intestinal barrier integrity by serving as an energy source for colonocytes and upregulating tight junction proteins such as occludin, claudin-1, and ZO-1 [84]. This reinforcement of epithelial junctions helps prevent microplastic-induced increases in intestinal permeability and reduces the translocation of endotoxins. Second, CFS exerts potent anti-inflammatory and antioxidant effects by inhibiting reactive oxygen species (ROS) generation, suppressing NF-κB activation, and promoting the secretion of anti-inflammatory cytokines such as IL-10, while simultaneously downregulating proinflammatory mediators including TNF-α, IL-6, and IL-1β. Third, CFS helps suppress pathogenic bacteria that proliferate as a result of microplastic-induced dysbiosis, thereby supporting the restoration of a healthier microbial ecosystem [112]. CFS metabolites also modulate immune cell differentiation and signaling pathways by enhancing Treg activity and reducing Th17-mediated inflammation, both of which are essential for controlling chronic gut inflammation. Additionally, antioxidant molecules present in CFS neutralize microplastic-induced oxidative stress, preventing DNA damage and cellular injury in intestinal epithelial cells [113]. Overall, the bacterial CFS provides a safe, stable, and effective postbiotic strategy to mitigate microplastic-induced IBD. By restoring epithelial barrier function, modulating immune responses, reducing oxidative and inflammatory stress, and maintaining microbial homeostasis, CFS offers therapeutic benefits comparable to probiotics but without the risks associated with administering live microorganisms. Future studies are expected to identify the specific bioactive compounds responsible for these effects and to optimize CFS formulations for clinical applications targeting environmentally triggered intestinal disorders such as IBD [114].

### 6.5. Cell Wall Composition

Numerous components of bacterial cell walls, including peptidoglycan and LTA, possess intrinsic immunogenic properties capable of inducing specific host immune responses. LTA is a major surface-associated molecule of Gram-positive bacteria and can be spontaneously released into the extracellular environment, where it plays key roles in bacterial physiology and host–microbe interactions [115]. Experimental studies have demonstrated that LTA exhibits potent immunostimulatory activity. Local administration of LTA enhances nonspecific immune defenses and promotes the secretion of antimicrobial peptides such as human β-defensins and cathepsins [116,117]. In murine models of colitis, LTA treatment significantly ameliorated disease manifestations, improving clinical scores, weight gain, and external signs of inflammation [118]. Peptidoglycan, another crucial bacterial cell-wall component, is a large polymer essential for maintaining cellular morphology, structural integrity, and viability in both Gram-positive and Gram-negative bacteria. It also plays an important role in maintaining normal immune cell function in vivo. Peptidoglycan fragments can activate nucleotide-binding oligomerization domain (NOD1) signaling in phagocytic cells, influencing their maturation and antimicrobial activity [119]. Furthermore, in macrophage-like cell models stimulated with bacterial lipopolysaccharide, peptidoglycan derived from various *Lactobacillus* species has been shown to suppress the release of pro-inflammatory cytokines.

### 6.6. Bacterial Lysates

Bacterial lysate mixtures of non-viable bacterial components generated through mechanical or chemical lysis contain a diverse range of immunologically active molecules, including cell-wall fragments, LPS, peptidoglycans, DNA, RNA, and various bacterial metabolites. Although non-replicating, they retain the ability to interact with host immune and epithelial cells in beneficial ways. A key function of bacterial lysates is their ability to “train” the immune system to respond more efficiently and tolerantly to microbial stimuli [120]. By engaging pattern recognition receptors (PRRs), such as TLRs and NOD-like receptors (NLRs), they promote a balanced immune response stimulating protective innate immunity while preventing excessive inflammatory activation [121].

In the context of microplastic-induced IBD, bacterial lysates can counteract multiple pathological processes. First, they enhance the production of anti-inflammatory cytokines including IL-10 and TGF-β, while suppressing the overexpression of pro-inflammatory mediators such as TNF-α, IL-1β, and IL-6. Second, bacterial lysates reinforce the intestinal epithelial barrier by upregulating tight junction proteins (occludin, claudin, and ZO-1) and stimulating mucin secretion, thereby limiting microplastic-induced permeability and endotoxin translocation [122]. Third, they help partially restore gut microbial balance by promoting an environment conducive to the growth of beneficial bacteria, thereby mitigating MPs-induced dysbiosis. Additionally, several studies suggest that bacterial lysates reduce oxidative stress and modulate immune cell populations by enhancing Treg activity and limiting the recruitment of inflammatory macrophages and neutrophils [123].

### 6.7. EPS

EPSs are carbohydrate polymers located outside bacterial cells and are commonly associated with the cell surface. They are often secreted into the surrounding environment in a viscous, slime-like matrix composed of diverse organic and inorganic components, including phosphates, sulfates, acetyl groups, and acetic acid [124]. Currently, EPSs are widely utilized for their gelling capacity, thickening properties, emulsifying functions, and metal biosorption abilities [125]. Recent studies have demonstrated that EPSs also strengthen the intestinal barrier and attenuate GI inflammation. EPSs derived from *Lactobacillus plantarum* enhance mucosal homeostasis in the colon through activation of the c-Jun/Muc2 signaling pathway and promote epithelial cell differentiation. Moreover, EPS exhibit potent immunomodulatory activity [126]. In in vitro models, EPS from *L. plantarum* increased nitric oxide production, enhanced macrophage phagocytic activity, stimulated lymphocyte proliferation, and elevated IgA levels in the intestinal mucosa [127]. Bacterial extracellular vesicles (EVs) are spherical, lipid bilayer-enclosed structures secreted by symbiotic bacteria, enabling the horizontal transfer of genetic material among microbial species. Gram-negative bacteria produce outer membrane vesicles (OMVs), whereas Gram-positive bacteria release membrane vesicles (MVs). Both types of vesicles contain functional components derived from their parent bacteria, including polysaccharides, glycolipids, proteins, DNA, and RNA [128]. These bioactive molecules contribute to regulating intestinal barrier permeability, modulating intracellular signaling pathways, maintaining intestinal homeostasis, improving lipid metabolism, and facilitating gut–brain communication [129]. Studies show that EVs derived from *Clostridium butyricum* alleviate DSS-induced colitis by modulating M2 macrophage polarization and reshaping gut microbiota composition. Similar microbiota-regulating and anti-inflammatory effects have been reported for EVs produced by *Escherichia coli* and *Lactobacillus plantarum* [88].

## 7. Limitations and Future Perspectives

Despite their promising therapeutic potential, postbiotics face several limitations and challenges in addressing microplastic-induced IBD. A major limitation is the lack of standardized definitions, formulations, and dosages, which makes it difficult to compare findings across studies and translate experimental results into clinical applications. Moreover, the complexity of gut–microplastic interactions, along with inter-individual variability in gut microbiota composition, can significantly influence the therapeutic efficacy of postbiotic interventions [130]. Most available evidence is derived from in vitro or animal models, and human clinical trials remain limited, leaving uncertainties regarding safety, optimal delivery strategies, and long-term outcomes. Nevertheless, the future outlook for postbiotics in this context is highly encouraging. Advances in microbiome science, metabolomics, and nanotechnology are expected to deepen our understanding of specific postbiotic molecules and elucidate their mechanisms of action in mitigating microplastic-induced gut inflammation [131]. The development of targeted postbiotic formulations such as encapsulated SCFAs or engineered microbial metabolites may enhance stability, bioavailability, and efficacy within the intestinal environment. In the long term, combining postbiotics with probiotics or prebiotics in “synbiotic” formulations may offer an integrated approach for restoring gut homeostasis, reducing chronic inflammation, and protecting the intestinal ecosystem from the adverse effects of microplastic exposure [132].

## 8. Conclusions

Microplastics have emerged as concerning environmental contaminants capable of disrupting intestinal homeostasis, impairing epithelial barrier integrity, and triggering the chronic inflammation characteristic of IBD. Increasing evidence demonstrates that MPs promote gut dysbiosis, oxidative stress, immune dysregulation, and the activation of pro-inflammatory signaling pathways including NF-κB, MAPK, TLR4, and AhR which collectively contribute to the onset and progression of IBD. Postbiotics mitigate MP-induced intestinal damage through multiple protective mechanisms, including reinforcement of tight junctions, enhancement of mucus production, modulation of immune responses, suppression of inflammatory cytokines, and restoration of microbial balance. Key postbiotic compounds such as SCFAs, bacteriocins, EPS, tryptophan-derived metabolites, and CFS exert strong anti-inflammatory, antioxidant, and immunomodulatory activities that help maintain gut homeostasis under microplastic stress. Given their stability, safety profile, and broad range of beneficial actions, postbiotics represent a compelling therapeutic alternative or complement to probiotics in managing microplastic-induced IBD. Future research should aim to clarify specific molecular interactions, optimize targeted formulations, and validate clinical efficacy in humans to fully harness the therapeutic potential of postbiotics for preventing and treating microplastic-associated intestinal disorders.

## Figures and Tables

**Figure 1:**
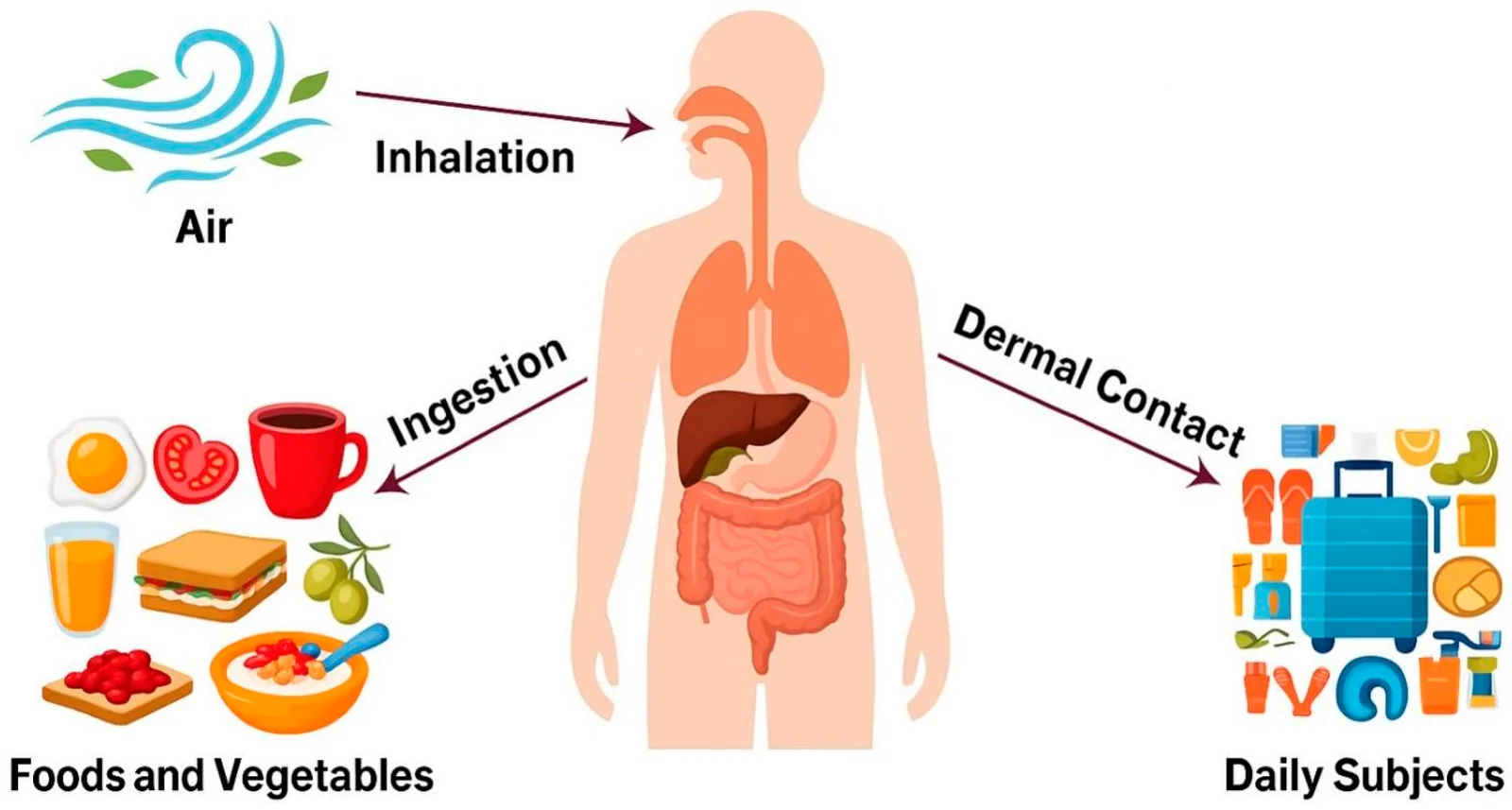
Revealing the pathways through which MPs enter the human body.

**Figure 2:**
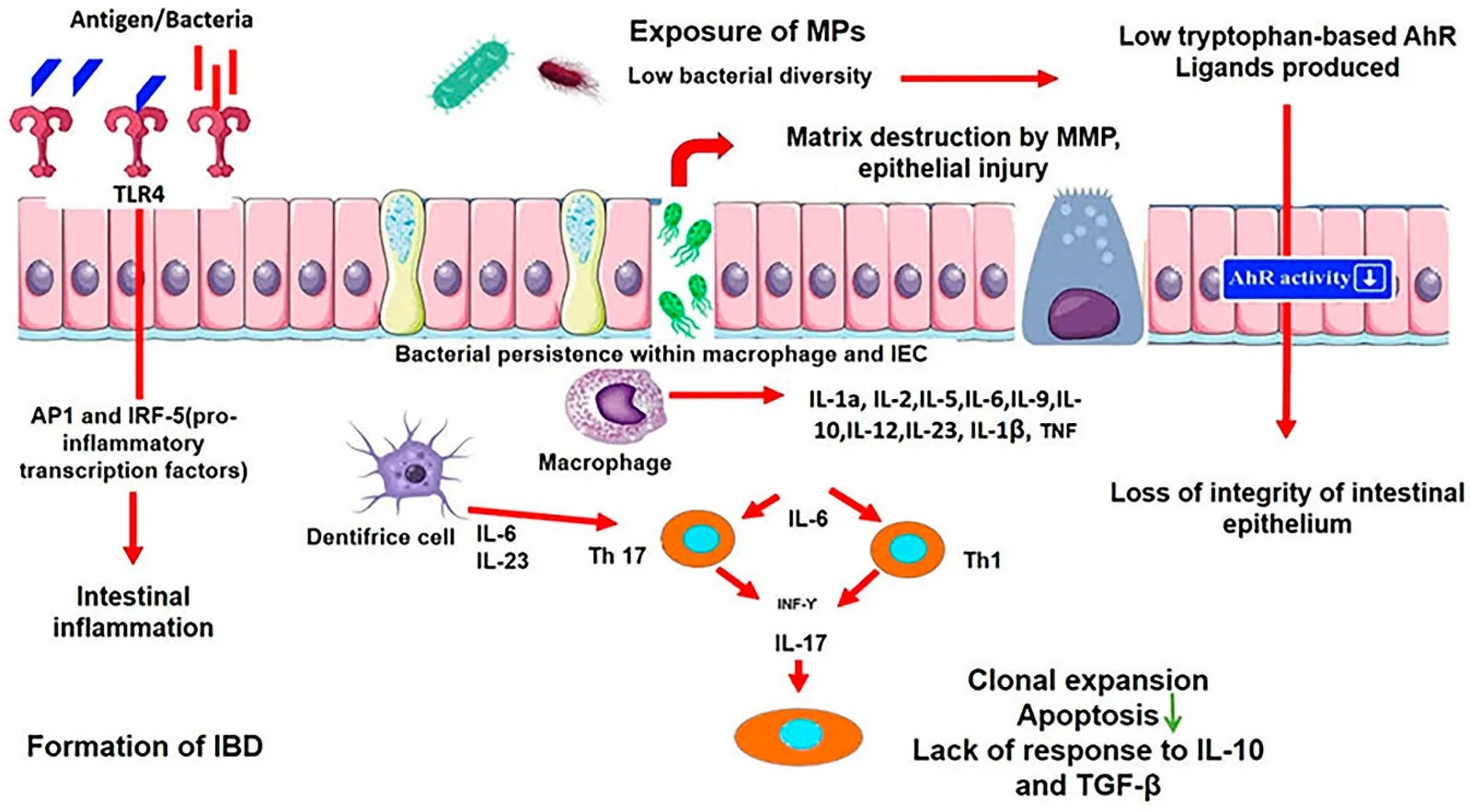
Schematic representation and signaling cascade of MPs in the intestinal epithelial cells for the development of IBD. Intestinal epithelial cells with exposure to MPs and formation of IBD. Polyethylene microplastic exposure reduces bacterial diversity and decreases the production of tryptophan-derived AhR ligands. Low AhR activation weakens epithelial integrity. Microplastics trigger inflammatory signaling through TLRs, increasing AP-1 and IRF-5 transcription factors and promoting intestinal inflammation. Epithelial damage and MMP-mediated matrix destruction allow bacteria to persist within macrophages and enterocytes, activating Th1 and Th17 pathways. Elevated proinflammatory cytokines contribute to chronic inflammation, apoptosis resistance, impaired TGF-β/IL-10 signaling, and clonal expansion of pathogenic T cells. Together, these disruptions lead to the initiation and progression of IBD.

**Figure 3:**
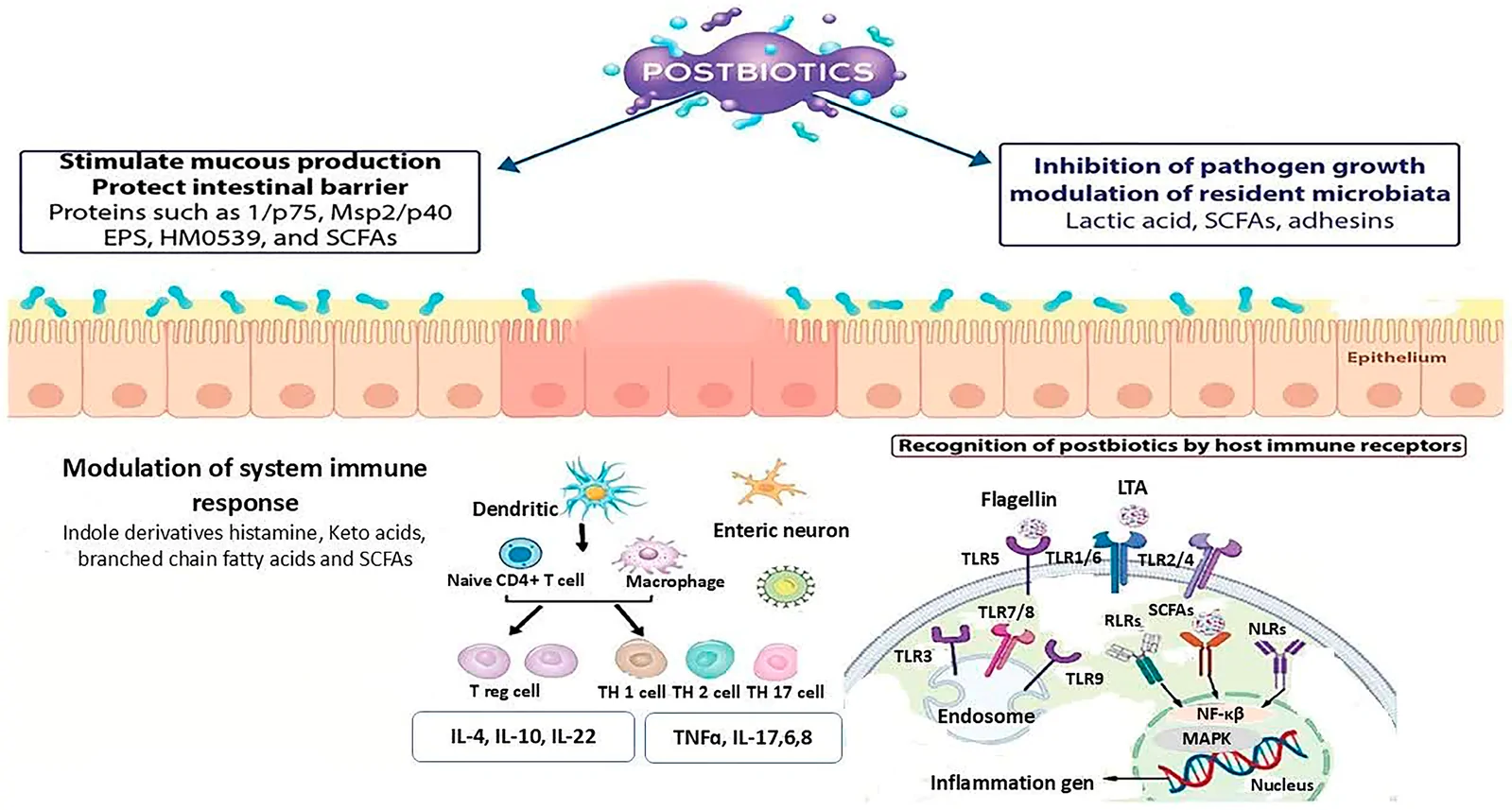
Mechanism of postbiotics in the treatment of IBD.

**Table 1:** Studies on the treatment of IBD with postbiotics.

Probiotics	Postbiotics	Type of Study	Dose	Duration of Treatment	Results	References
*Bifidobacterium adolescentis*	Cell-free Supernatant	Dextran sulfate sodium (DSS) induced colitis mice	0.2 mL	One week	Anti-inflammatory, antioxidant activity, and antibacterial properties	[80]
*Lactobacillus paracasei*	Extracellular vesicles	DSS-induced colitis mice	-	0–12 days	Attenuate the intestinal inflammatory response by enhancing the endoplasmic reticulum stress pathway	[81]
*Bifidobacterium bifidum*	Heat-killed cells	DSS-induced colitis mice	-	-	Anti-inflammatory effects and modulation of the gut microbiota	[82]
*Lactobacillus plantarum*	Heat-killed cells	DSS-induced colitis mice	1 mL	42 days	Enhance immune response and promote expression of tight junction proteins	[83]
*Lactobacillus plantarum*, and *Bifidobacterium lactis*	Cell-free supernatant	Indomethacin-induced colitis mice	10 mg/kg	5 days	Downregulate inflammatory responses and reduce oxidative stress	[84]
Gut microbiota from neonatal dairy calves	Ursodeoxycholic Acid	*Escherichia coli*-infected colitis neonatal mice	5.0 mg/mL	21 days	Inhibit bacterial growth, and alleviate dysbiosis of commensal bacteria through modulation of the TGR5-NF-κB axis	[85]
*Lactobacillus* species	Cell wall contents	Lipopolysaccharide-induced colitis rats	100 μg/animal	3 days	Limit immune-mediated inflammation and oxidative stress	[86]
*Limosilactobacillus fermentum*	Heat-killed cells	DSS-induced colitis mice	-	-	Alleviate intestinal barrier damage and gut microbiota disruption	[87]
*Lactobacillus plantarum*	Extracellular vesicles	DSS-induced colitis mice	1 mg/kg body weight	18 days	Modulate the gut microbiota	[88]
*-*	Butyrate	Patients with ulcerative colitis	60 mL	8 Weeks	Butyrate treatment for 4 and 8 weeks resulted in a significant reduction in the number of macrophages that were positive for nuclear translocated NF-kappaB.	[89]
-	SCFAs	In vitro (Human neutrophils)	30 mmol/L	24 h	SCFAs decreased IL-6 protein release from organ cultures.	[90]
*-*	Butyrate	Patients with ulcerative colitis	100 mL	20 days	A significant increase in the colonic IL-10/IL-12 ratio was found in butyrate-treated patients	[91]

## Data Availability

No new data were created or analyzed in this study. Data sharing is not applicable to this article.
